# Differential Expression of *OCT4* Pseudogenes in Pluripotent and Tumor Cell Lines

**DOI:** 10.22074/cellj.2016.3984

**Published:** 2016-04-04

**Authors:** Ensieh M. Poursani, Bahram Mohammad Soltani, Seyed Javad Mowla

**Affiliations:** Department of Molecular Genetics, Faculty of Biological Sciences, Tarbiat Modares University, Tehran, Iran

**Keywords:** OCT4 Pseudogenes, Stem Cell, Cancers, miR-145

## Abstract

**Objective:**

The human *OCT4* gene, the most important pluripotency marker, can generate at least three different transcripts (OCT4A, OCT4B, and OCT4B1) by alternative splicing. OCT4A is the main isoform responsible for the stemness property of embryonic stem (ES) cells. There also exist eight processed *OCT4* pseudogenes in the human genome with high homology to the OCT4A, some of which are transcribed in various cancers. Recent conflicting reports on *OCT4* expression in tumor cells and tissues emphasize the need to discriminate the expression of OCT4A from other variants as well as *OCT4* pseudogenes.

**Materials and Methods:**

In this experimental study, DNA sequencing confirmed the authenticity of transcripts of *OCT4* pseudogenes and their expression patterns were investigated in a panel of different human cell lines by reverse transcription-polymerase chain reaction (RT-PCR).

**Results:**

Differential expression of *OCT4* pseudogenes in various human cancer and pluripotent cell lines was observed. Moreover, the expression pattern of *OCT4*-pseudogene 3 (*OCT4-pg3*) followed that of OCT4A during neural differentiation of the pluripotent cell line of NTERA-2 (NT2). Although *OCT4-pg3* was highly expressed in undifferentiated NT2 cells, its expression was rapidly down-regulated upon induction of neural differentiation. Analysis of protein expression of OCT4A, *OCT4-pg1*, *OCT4-pg3*, and *OCT4-pg4* by Western blotting indicated that *OCT4* pseudogenes cannot produce stable proteins. Consistent with a newly proposed competitive role of pseudogene microRNA docking sites, we detected miR-145 binding sites on all transcripts of *OCT4* and *OCT4* pseudogenes.

**Conclusion:**

Our study suggests a potential coding-independent function for *OCT4* pseudogenes during differentiation or tumorigenesis.

## Introduction

*OCT4*, an important transcription factor in embryonic stem (ES) and embryonic carcinoma (EC) cells, has a crucial role in maintenance of pluripotency of stem cells and also in generating induced pluripotent stem (iPS) cells (1,3). There are three known variants of *OCT4 (OCT4A, OCT4B* and *OCT4-B1*) which are generated by different promoters or alternative splicing (4,5). OCT4A is localized in the nucleus of ES, EC, cancer stem and germinal cells, and germ cell tumors where it functions as the main transcription factor to sustain pluripotency and self-renewal of the cells (6,10). On the other hand, OCT4B is primarily expressed in the cytoplasm of tumor cells, and is unable to maintain pluripotency of stem cells (6,11). Recent studies have demonstrated the existence of an internal ribosome entry site (IRES) for OCT4B, which can produce three isoforms (OCT4B-265, OCT4B-190 and OCT4B-164) by alternative translation initiation (12,13). OCT4A and OCT4B-265 isoforms have identical POUDNA binding domain and C-terminal transactivation domain (CTD), but their N-terminal transactivation domain is different. OCT4B-190 and OCT4B-164 isoforms have the same CTD, but they have lost the N-terminal domain and a part of the POU-DNA binding domain (6,14). The newly discovered variant, OCT4B1, is localized in the nucleus and cytoplasm of pluripotent and undifferentiated cells (5,15). This isoform is generated by retaining intron2 of the OCT4B transcript as a cryptic exon. It has an in-frame stop codon (TGA) within its cryptic exon and thus produces a truncated protein with an N-terminal domain similar to OCT4B-265 as well as a part of POU specific (POUs) domain (5). In addition to pluripotent cells, further studies have demonstrated OCT4B1 expression in bladder, gastric and colorectal tumors where it acts as an anti-apoptotic factor (16,18). Pseudogenes have been traditionally described as non-functional genes which originate from protein coding genes. Currently, up to 20,000 pseudogenes have been detected in the human genome (19). Contrary to previous perceptions, current studies have indicated that some of these pseudogenes are transcribed. Aside from the possibility of having a biological function, they may aslo cause falsepositive signals in gene expression experiments such as reverse transcription-polymerase chain reaction (RT-PCR). Based on recent studies, some pseudogenes (i.e. *PTEN-pg* and *OCT4-pg4*) act as microRNA decoys and thereby regulate the effects of microRNAs on the corresponding protein-coding genes (20). Moreover, some pseudogenes have roles in gene silencing and thus regulate the expression of their parental genes. On the other hand, some transcribed pseudogenes are translated to generate truncated proteins or antigenic peptides. All of these findings indicate that pseudogenes are not junk DNA and can have important roles within normal and abnormal cells (20,22). 

So far, seven pseudogenes have been discovered for the human *OCT4* gene by bioinformatics and experimental analyses. All of them have been shown to be processed and transcribed in various cancer cell lines and tissues (11). *OCT4*-pseudogenes of *OCT4-pg1, OCT4-pg3* and *OCT4-pg4* have very similar exon structures to OCT4A, and hence could wrongly be detected as OCT4A. 

In this study, we investigated the expression pattern of *OCT4-pg1, OCT4-pg2, OCT4-pg3, OCT4-pg4, OCT4-pg5, OCT4-pg6* and *OCT4-pg7* in different human pluripotent and cancer cell types. Moreover, we cloned the whole sequence of *OCT4-pg1, OCT4-pg3* and *OCT4-pg4* in eukaryotic expression vectors to perform functional analyses. Finally, the protein production of *OCT4pg1, OCT4-pg3* and *OCT4-pg4* were examined by Western blotting. 

## Materials and Methods

### Cell culture

In this experimental study, 17 human cell lines were mainly provided by Pasteur Institute of Iran and Research Institute of Avicenna, included two human embryonic carcinoma cell lines, one normal fibroblast cell line (HS-5) and HEK293 (embryonic kidney) and 13 human tumor cell lines, namely U87MG and A172 (glioblastoma), Daoy (medulloblastoma), 1321N1 (brain astrocytoma), Jurkat (T-Cell lymphoma), Y79 (retinoblastoma), PC3 (prostate adenocarcinoma), Raji (Burkit’s lymphoma), Ovcar3 (ovary adenocarcinoma), HepG2 (hepatocellular carcinoma), MCF7 (breast adenocarcinoma), 5637 (urinary bladder carcinoma), HeLa (cervix adenocarcinoma), and NT2 and NCCIT (pluripotent embryonic carcinoma). 

Jurkat, Raji, ovcar3, U87, 5637 and NCCIT were cultured in RPMI-1640 medium supplemented with 10% fetal bovin serum (FBS, Gibco, UK), penicillin (100 U/ml) and streptomycin (100 µg/ ml, Gibco, UK). The Y-79 cell line was cultured in RPMI-1640 medium supplemented with 20% FBS. HS5, HepG2, MCF7, NT2, HeLa, A172, Daoy and HEK293 cells were cultured in High Glucose Dulbecco’s Modified Eagle Medium (DMEM, Gibco, UK, 4500 mg/l) supplemented with 10% FBS, sodium pyruvate and penicillin/streptomycin as described above. All cell lines were incubated at 37˚C (humidified) and 5% CO_2_. 

The human NT2 cells (kindly provided by Dr. Peter Andrews at Sheffield University) was propagated in DMEM/F-12 (Invitrogen, Gaithersburg, MD) supplemented with 10% FBS and 1% penicillin/streptomycin, and incubated at 37˚C in 5% CO_2_. NT2 cells were treated with all-trans retinoic acid (RA, Sigma-Aldrich, Germany) to induce their differentiation into neural-like phenotype as described previously (16). Briefly, 2 days before RA induction, cells were seeded in six-well plates at a density of 3-4×10^4^in 2 ml growth medium per well. RA was added to the growth medium at a final concentration of 10 mM, and the differentiation medium was renewed twice a week. Cultured cells from three replicates at 0, 3, 7, 14 and 21 days after RA treatment were harvested for RNA extraction and subsequent RT-PCR experiments. 

### RNA extraction and cDNA synthesis

Total RNA was extracted using TRIzol (Invitrogen, UK) according to the manufacturer’s instruca tion. The quality and quantity of extracted RNA were examined by agarose gel electrophoresis and spectrophotometery respectively. All extracted RNA samples were treated with DNaseI (Fermentas, Lithuania) and incubated at 37˚C for 30 minn utes. The enzyme was then inactivated by the addition of Ethylenediaminetetraacetic acid (EDTA, 50 mM) and incubation at 65˚C for 10 minutes. Subsequently, 2 µg of each DNase-treated RNA was used to synthesize cDNA by using reverse transcriptase (Fermentase, Lithuania) and oligodT primers according to the manufacturer’s instruction. The efficiency of DNase treatment and lack of DNA contamination was tested by having a NoRT control in all of RT-PCR experiments. 

### Reverse transcription-polymerase chain reaction

RT-PCR analysis of *OCT4* pseudogenes was carried out using specific primers (Table 1) that can exclusively amplify each *OCT4* pseudogene transcript. Details of PCR conditions for each *OCT4* pseudogene is summarized in Table 1. The PCR program included an intial step of 95˚C for 4 minc utes, followed by 35 cycles of denaturation at 95˚C for 30 seconds, annealing for 30 seconds and extension at 72˚C for 45 seconds, and a final extenn sion step of 72˚C for 7 minutes. 

**Table 1:** Details of primers and PCR conditions for amplification of OCT4A, *OCT4* pseudogenes, and the internal control, GAPDH


Primer name	Primer sequence	Annealing temp	Amplicon size (bp)

*OCT4-Pg1*	F: 5´-CAT GCA RGC CCG AAA GAG AAA GCR AR-3´	60	107
	R: 5´-TGT GGC TGA TCT GCA GTG TGG G-3´		
*OCT4-Pg2*	F: 5´-GTG TAC ATG TTT ATA AAG TTT GTG GTA GTG TTC-3´	58	380
	R: 5´-GGG TCG CTA GGT AAT TTT GTC ACT GG-3´		
*OCT4-Pg3*	F: 5´-CTT CTC ACC CCC TCC AGG C-3´	62	841
	R: 5´-CCA CTG CTT GAT CGC TTG C-3´		
*OCT4-Pg4 *	F: 5´-GGG ACA CCT GGC TTC GGA TG-3´	56	172
	R: 5´-CCC CAC ACC TCA GAG CCT GA-3´		
*OCT4-Pg5*	F: 5´-CAG TGA TTA TGC ACC ATG AGA GGA-3´	56	179
	R: 5´ GGG AAA GGC ACT AAG GAA CAC AG-3´		
*OCT4-Pg6*	F: 5´-CCT AGC AAA ACC TCA ACG AGT CCC AG-3´	60	219
	R: 5´-CAA GAG GTT GTG GTG AGC GAA GG-3´		
*OCT4-Pg7*	F: 5´-GCC AAA TTG CTG AAG CAG AAG GAT ATC-3´	58	347
	R: 5´-CTG GTC TTG TGA AAG GAT ACT CAG TGG-3´		
*OCT4A*	F: 5-CTT CTC GCC CCC TCC AGG T-3´	65	495
	R: 5´-AAA TAG AAC CCC CAG GGT GAG C-3´		
*GAPDH*	F: 5-GCCACATCGCTCAGACAC-3´	58	140
	R: 5´-GGCAACAATATCCACTTTACCAG-3´		


PCR; Polymerase chain reaction.

#### Cloning and sequencing of transcribed *OCT4* pseudogenes

The PCR products were separated on a 1.5% agarose gel and the bands were excised and extracted from the gel using DNA extraction kit (GeneAll Biotechnology, South Korea) and then cloned into the PTZ57R/T vector (Fermentase, Lithuania). The specificity and authenticity of the amplicons was further confirmed by DNA sequencing (Applied Biosystems, South Korea). 

### Constructing expression cassettes for *OCT4-pg 1, 3* and *4*

Using Pfu enzyme (GeneAll Biotechnology, South Korea) and specific primers for flanking regions of *OCT4-pg1, OCT4-pg3* and *OCT4-pg4* on genomic DNA, we amplified the corresponding genomic DNA by PCR. Nested-PCR was then performed by specific primers for coding sequences of each pseudogene. PCR products were then extracted from agarose gel, cloned in a TA cloning vector (PTZ57R/T) and their authenticity confirmed by DNA sequencing. Next, the amplified segments of the pseudogenes were digested by the NotI restriction enzyme and then cloned in the PCMV6-Neo expression vector. The specificity of the sequences and correct direction of cloned fragments inside the vector were further confirmed by DNA sequencing. 

### Western blotting

Untransfected HeLa, 5637, 1321, A172, NCCIT and NT2 cell cultures were lysed with 1 ml of lysis buffer [1% Triton X-100, 5 mM EDTA, 50 mM TrisHC1, pH=7.4, 150 mM NaCl, 0.1% sodium dodecyl sulfate (SDS)], 1% protease inhibitor cocktail and phosphatase inhibitor (Sigma, USA). Protein concentrations were measured by the Bradford Protein Assay reagent. Briefly, 20 µg of cell lysates were isolated on 12.5% SDS-PAGE and transferred onto a Polyvinylidene difluoride (PVDF) membrane (Millipore, USA). Blocking was carried out with 5% skim milk in tris-buffered saline (Sigma-Aldrich, Germany), containing 0.05% Tween20 (TBS-T). The membrane was then incubated for 1 hour with primary mouse anti-Oct-3/4 sc-5279 antibody (Santa Cruz, USA) diluted in 3% blocking buffer, and washed with PBS for 30 minutes at room temperature. A secondary HRPconjugated sheep-anti-mouse antibody (Avicenna Research Institute, Iran) was added next and incubated at RT for 1 hour. After washing, signals were detected using the Amersham ECL Prime Western Blotting Detection kit (GE Healthcare Life Sciences) and quantified by Gel Logic 2200 (Kodak, Japan). 

### Cell cycle analysis

HeLa cells were transfected with *OCT4-pg1, OCT4-pg3,* and *OCT4-pg4* vectors separately, and collected 48 hours after transfection. Harvested cells were washed with phosphate buffered saline (PBS, Sigma-Aldrich, Germany) and fixed in 1 ml ice-cold 70% ethanol for 30 minutes. Cells were stained with 50 mg/ml of propidium iodide (PI) solution (SigmaAldrich) containing 0.1% Triton X-100 and 10 mg/ ml RNaseA (Takara, Japan), mixed well and incubated for 5 to 10 minutes at room temperature. Prepared cells were then analyzed by a flow cytometer instrument (Becton Dickinson Bioscience, San Jose, CA). 

## Results

### Differential expression pattern of *OCT4*-pseudogenes in different human cell types

**Fig.1:**
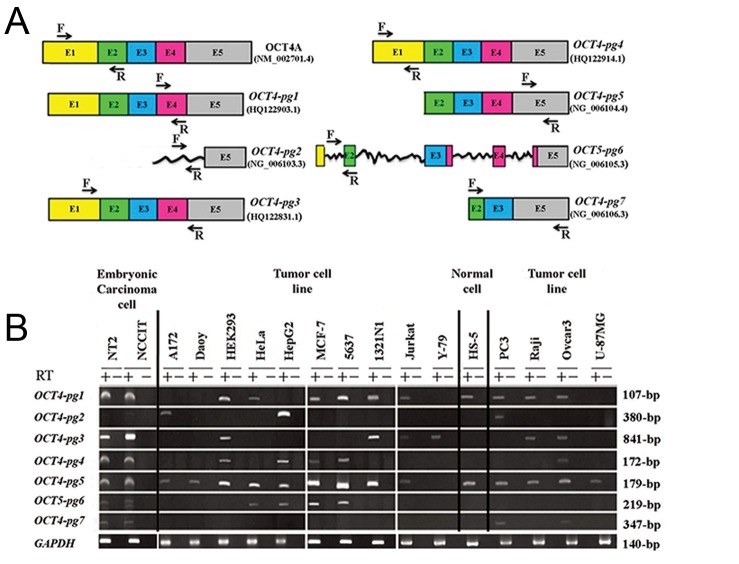
A. Schematic representation of *OCT4* pseudogenes. *OCT4-pg1, OCT4-pg3* and *OCT4-pg4* have highly similar nucleotide sequences to that of the OCT4A transcript. *OCT4-pg5* transcript lacks exon1, and *OCT4-pg7* lacks exon1, exon4, and part of exon2. *OCT4-pg2* has a part of exon5, and *OCT4-pg6* has all five exons, incompletely. Rough lines in *OCT4-pg2* and *OCT4-pg6* are remained sequences which are derived from *OCT4* introns and B. RT-PCR analysis of *OCT4*-pseudogenes in different human pluripotent and cancer cell lines by specific primer sets. GAPDH was used as an internal control. RT-PCR; Reverse transcriptase-polymerase chain reaction.

Based on RT-PCR data, *OCT4-Pg1* was detected in NT2 and NCCIT, HS-5, and nine cancer cell lines (A172, HEK293, MCF-7, 5637, 1231N1, Jurkat, PC3, Raji, and Ovcar3) (Fig.1A). In contrast, *OCT4-Pg2* expression was restricted only to A172, HepG2 and PC3 cells. *OCT4-Pg3* was expressed in HEK293, 1321N1, Jurkat, Y79, Raji and Ovcar3 but showed a significant expression in EC cells. *OCT4-Pg4* showed detectable signals in EC cells as well as in HEK293, HepG2, MCF-7, 5637 and Ovcar3 tumor cell lines. *OCT4-Pg5* was expressed in all cell lines except Y-79. Expression of *OCT4-Pg6* was detected in EC cells as well as in HeLa, HepG2, MCF-7 and 5637 cells. *OCT4-Pg7* was also detected in EC cells, and PC3 and Ovcar3 cell lines (Fig.1B). DNA sequencing confirmed authenticity of all amplified PCR products. 

### *OCT4-pg1, OCT4-pg3* and *OCT4-pg4* produce unstable proteins

**Fig.2:**
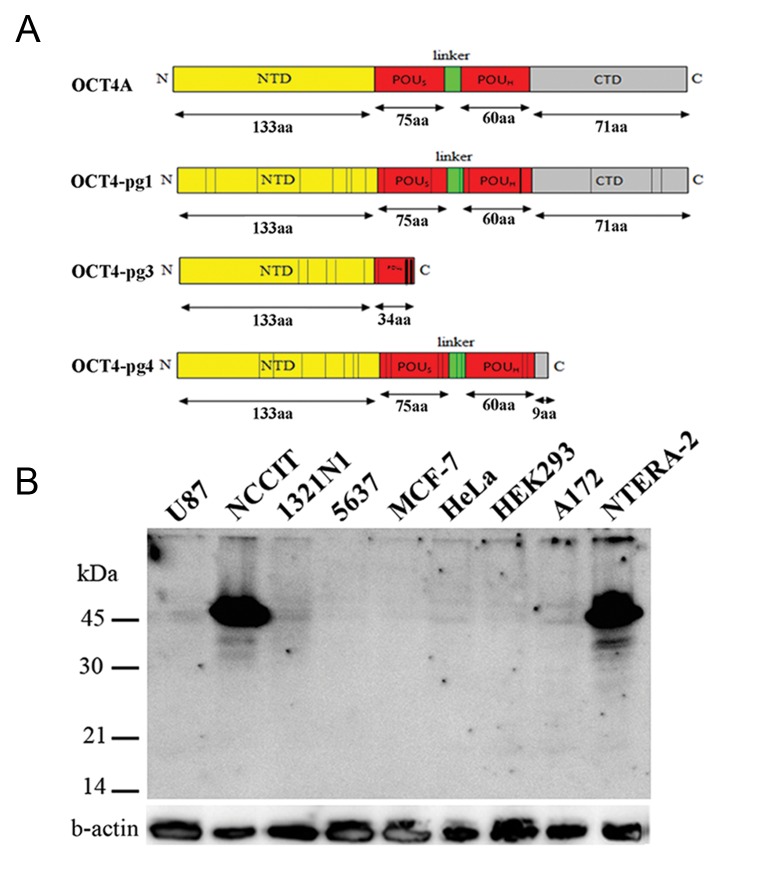
A. A schematic view of OCT4A protein structure, along with predicted protein structures of *OCT4-Pg1, OCT4-Pg3*, and *OCT4-Pg4*. The putative *OCT4-Pg1* protein is similar to OCT4A, containing intact NTD, POU domain and CTD. *OCT4-Pg3* can potentially produce a truncated protein with NTD and a part of the POUs domain. The predicted *OCT4-pg4* protein contains NTD and POU domain, but lacks a large part of the C-terminal domain. Vertical lines within the depicted structures of *OCT4* pseudogenes indicate the position of point mutations which have changed the amino acid sequences of the predicted proteins and B. Western blotting in different human cell types transfected with *OCT4-Pg1, OCT4-Pg3* and *OCT4-Pg4* expression vectors. The NCCIT and NT2 cell lines were used as positive controls for OCT4A, while U-87MG was used as a negative control for OCT4A and *OCT4* pseudogenes. Using the sc-5279 antibody, we detected OCT4A protein exclusively in NCCIT and NTERA-2 cell lines but did not detect *OCT4* pseudogenes in the cells which expressed them at the transcript level. Note that the internal control b-actin protein is detectable at similar intensities in all examined cell lines.

*OCT4-pg1, OCT4-pg3* and *OCT4-pg4* might potentially produce proteins. For instance, *OCT4-pg1* transcript can produce a protein similar to OCT4A, containing NTD, CTD and POU domain. Due to point mutations, *OCT4-pg3* encodes a truncated protein with a complete NTD and a partial POUs domain. Hypothetical *OCT4-pg4* protein misses a large part of CTD, but has intact NTD and POU domain. Therefore, we decided to experimentally examine the protein expression of these pseudogenes by Western blotting based on mouse anti-OCT3/4 sc-5279 monoclonal antibody (raised against amino acids 1-134 of OCT-3/4 of human origin), an antibody against NTD. Therefore, antibodies which recognize NTD of OCT4A can also detect *OCT4-pg1, OCT4-pg3* and *OCT4-pg4* but can be discriminated from OCT4A by size differences (Fig.2A). We used NT2 and NCCIT cells as positive controls, U-87MG as a negative control and six somatic cancer cell lines (A172, 5637, 1321N1, HeLa, HEK293, and MCF- 7) that express the *OCT4* pseudogenes. We detected a high level of OCT4A expression in NT2 and NCCIT cells, but no detectable signal was observed for Fig.1: A. Schematic representation of *OCT4* pseudogenes. *OCT4-pg1, OCT4-pg3* and *OCT4-pg4* have highly similar nucleotide sequences to that of the OCT4A transcript. *OCT4-pg5* transcript lacks exon1, and *OCT4-pg7* lacks exon1, exon4, and part of exon2. *OCT4-pg2* has a part of exon5, and *OCT4-pg6* has all five exons, incompletely. Rough lines in *OCT4-pg2* and *OCT4-pg6* are remained sequences which are derived from *OCT4* introns and B. RT-PCR analysis of *OCT4*-pseudogenes in different human pluripotent and cancer cell lines by specific primer sets. GAPDH was used as an internal control. RT-PCR; Reverse transcriptase-polymerase chain reaction. *OCT4* pseudogene potential proteins (Fig.2B).

### Cell cycle alteration following overexpression of *OCT4-pg1, OCT4-pg3* and *OCT4-pg4*

**Fig.3:**
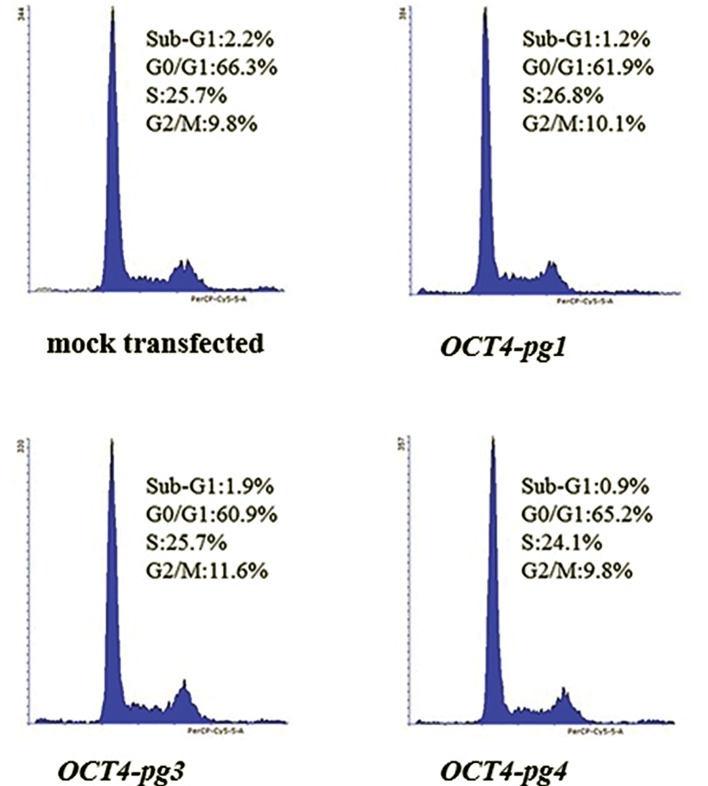
Cell cycle analysis of HeLa cells transfected with either the mock p-EGFPC-1 expression vector or the same vector containing the sequence corresponding to *OCT4-pg1, OCT4-pg 3* or *OCT4-pg4*.

To investigate the potential function of *OCT4-pg1, OCT4-pg3* and *OCT4-pg4*, we overexpressed them in HeLa cells. Cell cycle analysis was then undertaken after staining the DNA content of the cells with PI. Compared with the control cells transfected with mock PCMV6-Neo vector, the transfected cells demonstrated subtle decline in distribution of cells in the G1 and sub-G1 phases, and a slight elevation of distributed cells in the S phase of cell cycle (Fig.3). 

### Downregulation of *OCT4-pg3* during the course of neural differentiation of NT2 cells

**Fig.4:**
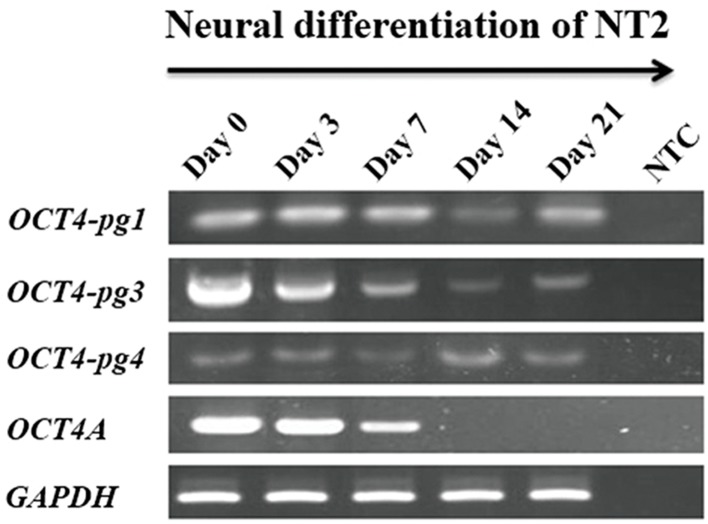
RT-PCR analysis of *OCT4-Pg1, OCT4-Pg3* and *OCT4-Pg4* expression during neural differentiation of NT2 cells. Note that *OCT4-Pg3* is expressed at high levels in undifferentiated cells but is down-regulated upon the initiation of differentiation. NTC; Non-template control and RT-PCR; Reverse transcriptase-polymerase chain reaction.

As demonstrated in Figure 4, *OCT4-pg3* was highly expressed in undifferentiated NT2 cells, and its expression is gradually diminished upon the induction of differentiation. The gene expression alteration of *OCT4-pg3* correlated with that of OCT4A, suggesting a similar regulatory control for both genes. A similar decline in the expression pattern was not observed for *OCT4-pg1* and *OCT4-pg4* (Fig.4).

### Conservation of miR-145 binding sites in OCT4A and *OCT4*-pseudogene sequences

Considering a newly proposed competing endogenous RNA (ceRNA) role for pseudogenes, we hypothesized a non-coding functional role for *OCT4* pesudogene transcripts of sponging a well-known inhibitor of *OCT4*, miR-145. We scanned the sequences of the *OCT4* pesudogenes to find potential conserved binding sites for miR-145. As shown in Table 2, the seed sequence of miR-145 exists on almost all *OCT4* pesudogenes.

**Table 2:** A sequence complementary pattern of OCT4 and OCT4-pgs with miR-145. The conserved nucleotides are marked with vertical lines between the two sequences. The seed sequence of miR-145 is highlighted by a green box. A conservation of the putative miR-145 binding site sequence is also provided for each pseudogene


Gene	Targetsiteofhsa-miR-145-5P	Location

*OCT4A*	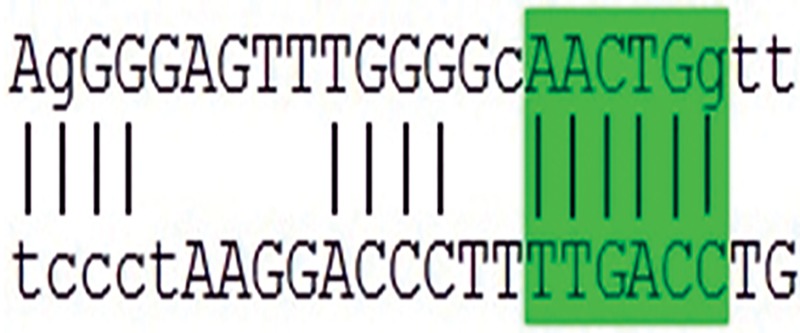	3´-UTR
*OCT4-pg1*	AAACTGAG	3´-UTR
*OCT4-pg2*	GAAACTGG	Exon5
*OCT4-pg3*	AGACTGGA	Exon1
*OCT4-pg4*	GAAACTGGA	Exon1
*OCT4-pg5*	AACTGG	3´-UTR
*OCT4-pg6*	AACTGG	Exon5
*OCT4-pg7*	GGGAAACTGG	3´-UTR


## Discussion

*OCT4* is a crucial transcription factor with a key role in maintaining the stemness state of pluripotent cells (8, 13, 23, 24). It was initially believed that *OCT4* is exclusively expressed in embryonic stem cells, however, its recent detection in some cancer cells and tissues ignited a dispute on the accuracy of the data (19, 25). A strong possible source for the conflicting reports on *OCT4* may be due to non-specific primers which were unable to discriminate OCT4A from its pseudogenes (26). In other words, RT-PCR artifacts and misdetection of the OCT4A isoform could be partly derived by the amplification of highly homologous *OCT4* pseudogenes at the transcript level (27). Therefore, using specific primers that can discriminate *OCT4* pseudogenes from the OCT4A variant is crucial for RT-PCR analysis of *OCT4* expression.

Considering the conflicting reports on expression analysis of OCT4A and *OCT4* pseudogenes in embryonic stem cells, and cancer cell lines and tissues, we evaluated here the expression pattern of all known *OCT4* pseudogenes in various human pluripotent and tumor cell types. Our data support the idea that misdetection of OCT4A in somatic cancer cell types may be caused by non-specific primers that could in addition amplify one or more *OCT4* pseudogenes. Moreover, our data revealed that different *OCT4* pseudogenes are differentially expressed in various tumor cell lines, suggesting a unique expression regulation for each of them. For instance, while *OCT4-pg2* is barely detectable in most examined cell lines, it showed a very high level of expression in HepG2 cells.

Since *OCT4* pseudogenes might have some functional activity at the transcript and/or protein levels (11), and are widely expressed in tumor cells and tissues, they might have a putative role in tumor cell proliferation or tumor progression (27). Our data demonstrated a lack of protein expression for pseudogenes of *OCT4*. However, the fact that *OCT4-pg3* expression is regulated during the course of neural differentiation of NT2 cells, suggest a functional association, albeit at the transcript level. Interestingly, a coding-independent competing role has already been proposed for some pseudogenes. Accordingly, a sponge role for *OCT4-pg4* in binding, and hence in releasing the inhibitory function of miR-145 is reported by Wang et al. (28). Given that we identified a conserved miR-145 binding site for almost all *OCT4* pseudogenes and considering the role of miR-145 as a tumor-suppressor gene (29), it would be plausible that the wide expression of *OCT4* pseudogenes in various cancer types may be associated with tumorigenesis. However, this hypothesis needs to be experimentally validated in different tumor cell lines.

## Conclusion

We show that *OCT4* pseudogenes are differentially expressed in various human pluripotent and tumor cell types. However, Western blotting revealed no protein expression for the *OCT4* pseudogenes. This suggests that these pseudogenes may have a potential non-coding function, possibly by having a sponging effect on miR-145. 
